# Ability of AZD1222 vaccination to elicit neutralising antibodies against SARS-CoV-2 VOC B.1.617.2 (Delta)

**DOI:** 10.1016/S0140-6736(21)01462-8

**Published:** 2021-06-28

**Authors:** Emma C Wall, Mary Wu, Ruth Harvey, Gavin Kelly, Scott Warchal, Chelsea Sawyer, Rodney Daniels, Lorin Adams, Philip Hobson, Emine Hatipoglu, Yenting Ngai, Saira Hussain, Karen Ambrose, Steve Hindmarsh, Rupert Beale, Andrew Riddell, Steve Gamblin, Michael Howell, George Kassiotis, Vincenzo Libri, Bryan Williams, Charles Swanton, Sonia Gandhi, David LV Bauer

**Affiliations:** 1 The Francis Crick Institute, 1 Midland Road, London, UK; 2 National Institute for Health Research (NIHR) University College London Hospitals (UCLH) Biomedical Research Centre and NIHR UCLH Clinical Research Facility, London, UK; 3 Worldwide Influenza Centre, The Francis Crick Institute, 1 Midland Road, London, UK; 4 University College London, Gower Street, London; 5 Department of Infectious Disease, St Mary’s Hospital, Imperial College London, London, UK; 6 Genotype-to-Phenotype UK National Virology Consortium (G2P-UK)

The B.1.617.2 ‘Delta’ variant of SARS-CoV-2 continues to drive a sharp increase in COVID-19 cases in the United Kingdom, with a current doubling time of 3.5-16 days^a^, consistent with previous pandemic waves during 2020-2021, and driving an sustained increase in the reproduction number (R) to 1.2-1.4^b^. Daily hospital admissions and the number of patients requiring mechanical ventilation are now increasing in both England and Scotland, despite the ongoing rollout of widespread vaccination in the United Kingdom^a^.

The ChAdOx1 nCoV-19 (AZD1222, Oxford-AstraZeneca) vaccine forms the core of the United Kingdom’s vaccination programme and the worldwide COVAXX programme. To determine B.1.617.2 sensitivity to AZD1222-induced neutralising antibodies (NAbs) and to compare this to our previous measurements of BNT162b2 (Pfizer-BioNTech)-induced NAbs (Wall, Wu et al., Lancet 2021)^1^, we carried out a second initial analysis of Legacy study participants vaccinated with AZD1222. Legacy was initiated in early 2021 by University College London Hospitals and the Francis Crick Institute to track serological responses to vaccination during the national COVID-19 vaccination programme in prospectively recruited healthy staff volunteers. A description of the methods and clinical cohort, are available in the appendix. The Legacy study was approved by London Camden and Kings Cross Health Research Authority Research and Ethics committee (IRAS number 286469) and is sponsored by University College London Hospitals.

Using a high-throughput live-virus SARS-CoV-2 neutralisation assay, we determined NAb titres (NAbTs) in 106 participants (Table 1, median age 34 years, [IQR 29-42]) following either 1 dose (*n* = 50, median time after first dose = 41 days [IQR 30-51]) or 2 doses (*n* = 63, median time after second dose = 31 days [IQR 19.5-46], median interval between doses = 63 days [IQR 62-69.5]) of AZD1222, against five SARS-CoV-2 strains. Consistent with our previous report, we included a strain with the original spike sequence (‘Wildtype’), a D614G-containing strain isolated during the first UK wave of infection in 2020, and three VOCs: B.1.1.7 (‘Alpha’, first detected in Kent, England), B.1.351 (‘Beta’, first detected in South Africa), and B.1.617.2 (‘Delta’, first detected in India).

Two doses of AZD1222 generated NAb activity against the Wildtype strain bearing a spike identical to that encoded by the vaccine in all participants (median NAbT IC_50_ = 419), with a 2.1-fold [95%CI: 2.0-2.2] reduction in median NAbT relative to two doses of BNT162b2 (Pfizer-BioNTech) vaccine (Figure 1A). Moreover, median NAbTs against all SARS-CoV-2 variants were further reduced relative to BNT162b2: 2.4-fold [95%CI: 2.3-2.6] against D614G, 2.4-fold against B.1.1.7 [2.2-2.5], 2.5-fold [1.3-2.8] against B.1.351, and 2.5-fold [1.4-2.7] against B.1.617.2. Given the low responses against the latter two VOCs, we found that stratification of NAbTs into three groups (IC50 low [<40], medium [40–256], high [>256]) was most illustrative: While nearly all participants had a quantifiable NAbT against the D614G and B.1.1.7 variants (55/63, 87% [95%CI: 76%-94%], Figure 1B), significantly fewer participants had quantifiable NAbTs against B.1.351 and B.1.617.2 VOCs following two doses of AZD1222 (38/63, 60% [47%-72%]; and 39/63, 62% [49%-74%]; respectively, relative to the former 2 variants, X^2^ test p<0.0011). This contrasts strongly with our previous results where over 95% of participants had quantifiable NAbTs against B.1.351 and B.1.617.2 following two doses of BNT162b2 (189/195, 97%; and 186/195, 95%; respectively). Analysis of these data by ordered logistic regression confirmed vaccine type was associated with decreased NAbTs, independent of SARS-CoV-2 strain, in 2-dose vaccine recipients (p=0.0017, Table 2).

A single dose of AZD1222 generated a broad range of NAb activity in participants against Wildtype SARS-CoV-2 (Figure 1C). Given reports of enhanced NAb responses to VOCs B.1.1.7 and B.1.351 in individuals with, than without prior SARS-CoV-2 infection after a single dose of mRNA vaccines (Stamatatos et al., Science, 2021; Reynolds et al., Science, 2021)^2,3^, in the absence of concrete evidence of prior infection, we stratified NAbT by whether participants reported prior COVID-19 symptoms and found markedly different responses: those with prior COVID-19 symptoms (16/50, 32%) had significantly higher NAbTs against all strains than those without prior COVID symptoms after a single AZD1222 dose (5.1×10^-5^ ≤ p ≤ 3.1×10^-4^). Since many responses lay outside of the quantitative limit of detection, stratification of NAbTs was again informative: while participants without prior COVID-19 symptoms mostly (31/34, 91% [95%CI: 75%-98%]) had quantifiable NAbTs against Wildtype, significantly more NAb responses below the limit of detection against VOCs: 22/34 (65% [95%CI: 46%-80%]) against B.1.1.7; 30/34 (88% [72%-96%]) against B.1.351; and 29/34 (85% [68%-94%]) against B.1.617.2 (2.8×10^-10^ ≤ p ≤ 6.0×10^-6^, Figure 1D). Analysis by ordered logistic regression confirmed prior COVID-19 symptoms were associated with increased NAbTs, independent of SARS-CoV-2 strain, in single-dose AZD1222 recipients (p=0.0016, Table 2).

Together, our data here and previously-reported (Figure 2, Wall, Wu et al., Lancet, 2021)^1^ reveal that AZD1222 recipients have lower NAbTs than BNT162b2 recipients against SARS-CoV-2 variants, including B.1.617.2. This finding is in line with the vaccine-induced NAbTs observed during clinical trials of AZD1222 (Folegatti et al., Lancet, 2020)^4^ and BNT162b2 (Sahin et al., Nature, 2020)^5^. Notably, our data are consistent with preliminary observational estimates based on rates of S-gene target failure during PCR testing in England (Lopez-Bernal et al., medRxiv, 2021)^6^ and more recent data from Scotland (Sheikh et al., Lancet, 2021)^7^, which reports 19% reduced AZD1222 efficacy following 2 doses (60%) relative to 2 doses of BNT162b2 (79%) against the B.1.617.2 variant and similar to reduced efficacy against the B.1.1.7 variant following 2 doses (73% for AZD1222 vs. 92% for BNT162b2). The combination of these observational data with our laboratory data suggests that the correlation between NAbTs and vaccine efficacy in recent models (Khoury et al., Nature Medicine, 2021)^8^ continues to perform well across different vaccine types and SARS-CoV-2 variants (Figure 3). It further highlights that the lower starting NAbTs of AZD1222 recipients will now render vaccine efficacy more susceptible to any possible individual-level variation (e.g. prior infection, age, immune status, antibody durability, comorbidities). It should also be noted, however, that prevention of infection appears to require substantially higher NAbTs than prevention of the most severe COVID-19 and death. Therefore, although reduced in vitro neutralisation of VOCs predicts reduced AZD1222 vaccine efficacy against symptomatic infection with the same VOCs, close monitoring of the unfolding pandemic will reveal the extent to which the link with severe or fatal COVID-19 has been broken by all current vaccines.

Given our previously-reported observation of decreased NAbTs in older BNT162b2 recipients (Wall, Wu et al., Lancet, 2021)^1^, it is worth noting that our observation here of ~2.5-fold lower median NAbTs in 2-dose AZD1222 recipients relative to 2-dose BNT162b2 recipients is confounded by the fact that the AZD1222 cohort is significantly younger than the BNT162b2 cohort (median age = 33 years, [IQR 28-41] vs. median age = 42 years [IQR 33-52], p=2.3×10^-8^); comparison of 2-dose AZD1222 recipients to a more similar subset of the 2-dose BNT162b2 cohort (n=58, single study site, age < 50 years, dosing interval >40 days) (Table 2), shows a more pronounced reduction in median NAbTs against B.1.617.2 between 2-dose AZD1222 and 2-dose BNT162b2 recipients (Figure 3). Further serological examination of AZD1222 recipients will be needed as the UK vaccination programme continues, to assess the extent to which variables such as age affect NAbTs (especially beyond the median 24 days post-second dose examined here) and vaccine efficacy — along with increased standardisation across serological laboratories and refined correlates of protection against all SARS-CoV-2 variants.

Our data reinforce the need to recognise the increase in protection offered by a second vaccine dose, in the face of increasing COVID-19 cases driven by the B.1.617.2 variant. They also suggest that further booster immunisations will likely be needed, especially for more vulnerable groups that have received vaccines that induce lower-than-average NAbTs. As with mRNA vaccines, it may be feasible to prioritise the use of the ChAdOx1 vaccine, in light of severely restricted supply, according to confirmed prior COVID-19 exposure. Overall, our findings highlight the urgent need for expanded serological monitoring of NAbTs within sub-populations to better understand the evolution of vaccine efficacy and to facilitate the production of updated vaccines, in order to ensure maximum protection against SARS-CoV-2 variants.

## Supplementary Material

Appendix

## Figures and Tables

**Figure 1:**
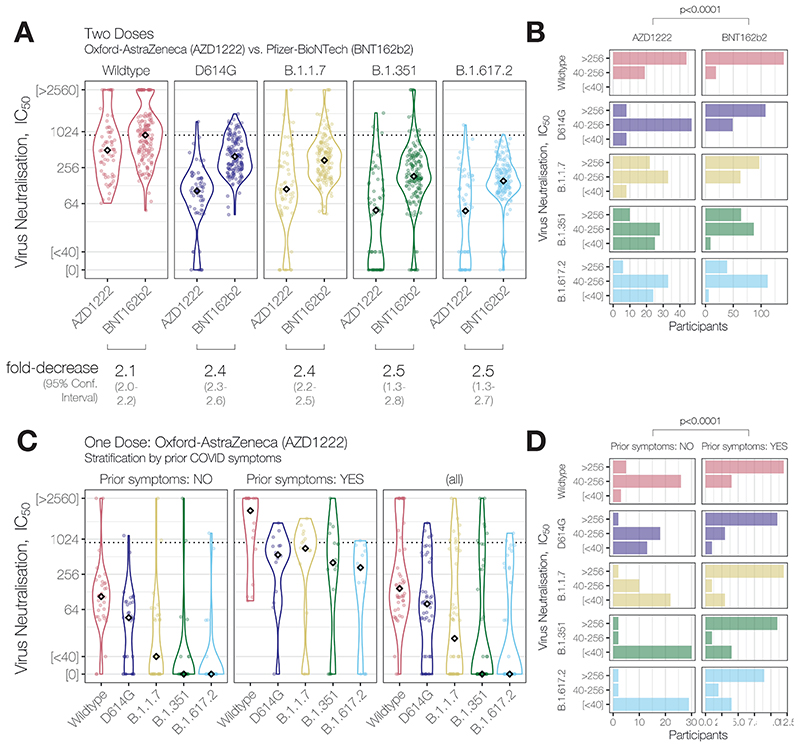
Neutralising antibody activity against SARS-CoV-2 variants of concern B.1.617.2 and B.1.351 elicited by partial or full vaccination with ChAdOx1 nCoV-19 (AZD1222, Oxford-Astra-Zeneca) and effect of reported prior COVID symptoms. (**A**) Neutralising antibody titres (NAbTs) against five SARS-CoV-2 strains from 63 study participants who had received 2 doses of ChAdOx1, comparised to 159 participants who had received 2 doses of BNT162b2. NAbTs are expressed as serum fold-dilution required to achieve 50% virus neutralisation (IC_50_), and shown (**B**) grouped into 3 response levels. (**C**) NAbTs from 50 participants following 1 dose of AZD1222, stratified according to participants’ report of prior COVID symptoms, and (**D**) grouped into 3 response levels.

**Figure 2:**
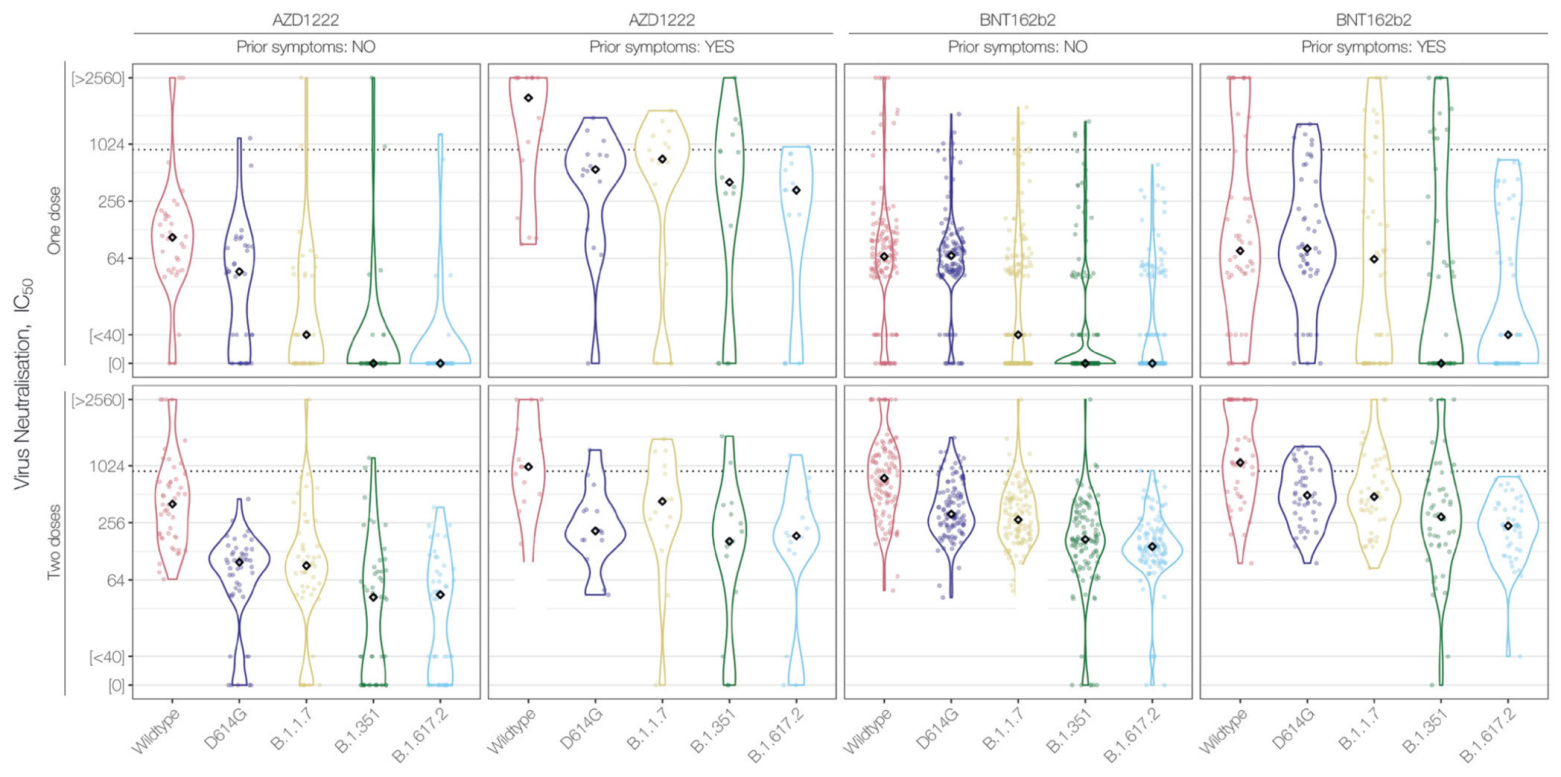
NAbT responses stratified by Vaccine Type, number of doses, and self-reported prior COVID symptoms. Data from this study and from (Wall, Wu, et al., Lancet, 2021).

**Figure 3:**
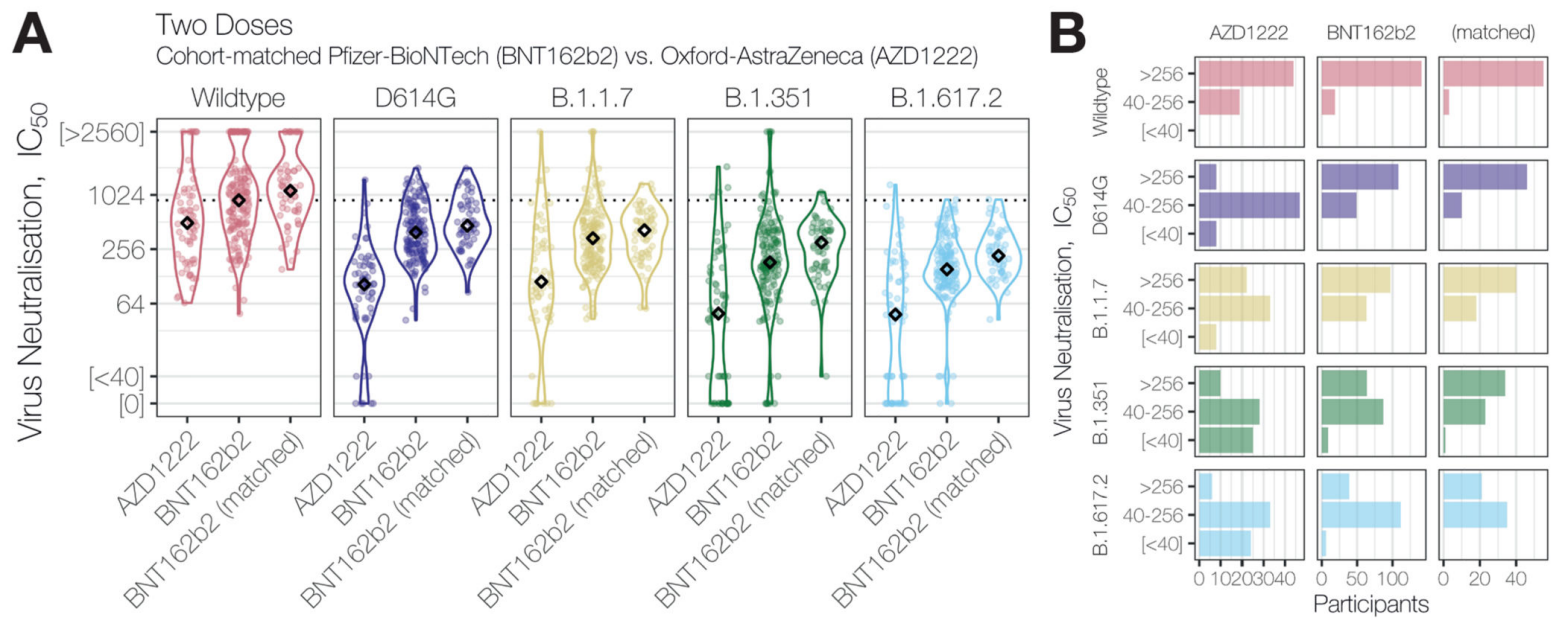
Neutralising antibody responses of ChAdOx1 recipients vs. cohort-matched BNT162b2 recipients. (**A**) Neutralising antibody titres (NAbTs) against five SARS-CoV-2 strains from study participants who had received 2 doses of ChAdOx1, comparised to participants who had received 2 doses of BNT162b2, and a matched subset of BNT162b2 recipients selected to match characterisitcs of ChAdOx1 chort (single study site, age<50 years, dose interval > 40 days), see Table 2. NAbTs are expressed as serum fold-dilution required to achieve 50% virus neutralisation (IC_50_), and shown (**B**) grouped into 3 response levels.

**Figure 4:**
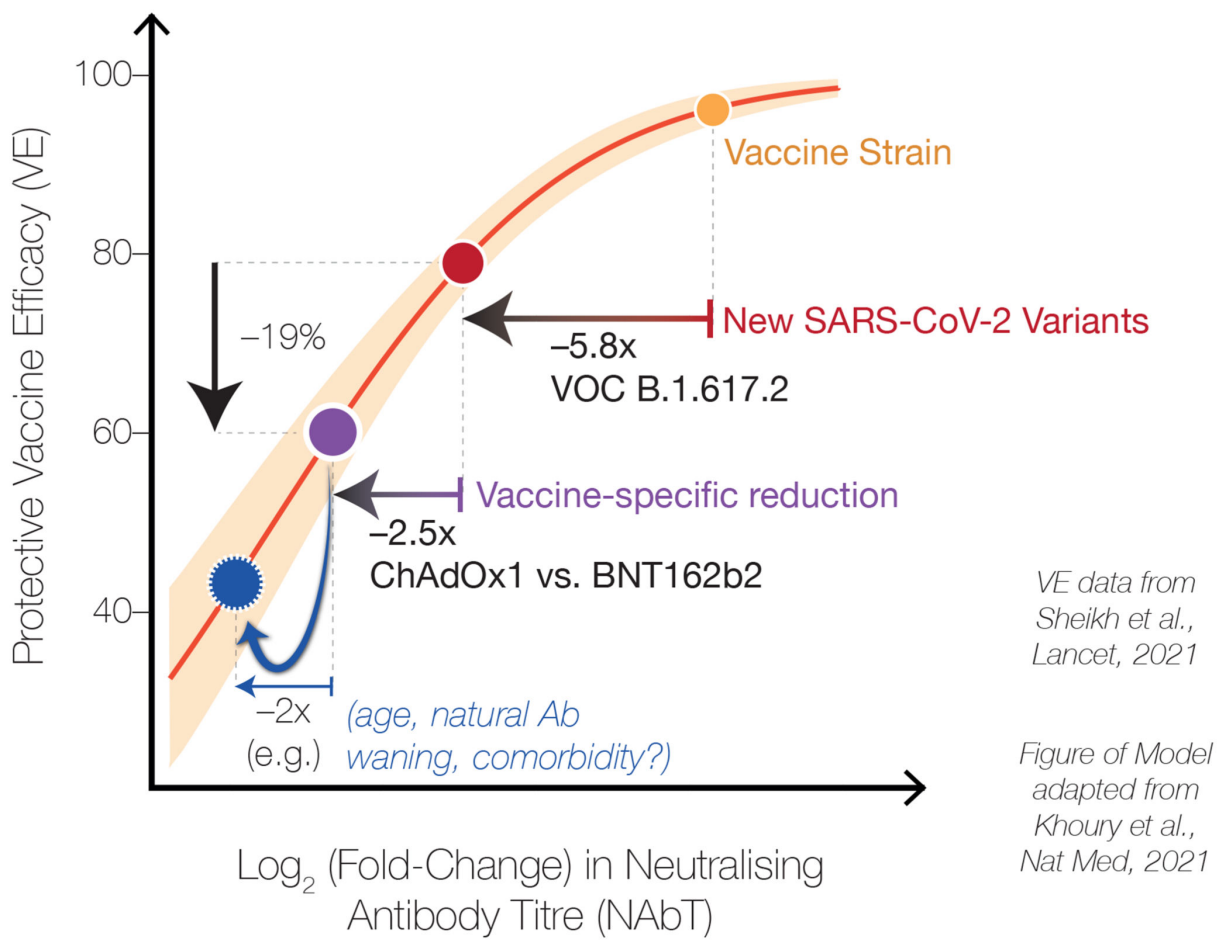
Schematic illustrating correlates between neutralising antibody titres against SARS-CoV-2 and vaccine efficacy (VE). Schematic based on the model of Khoury et al., overlayed with our laboratory measurements of neutralising antibody titres (NAbTs) against SARS-CoV-2 and observed real-world VE data from Sheikh et al., illustrating the relationship between NAbTs and VE. When NAbTs begin at a high level (e.g. against variants with spike proteins similar to the Wild-type spike in first-generation vaccines), small changes in NAbsTs have a small effect on VE. However, when titres begin from a lower level, such as from a cumulative effect of VOCs and vaccine type, small additional changes in NAbTs (e.g. due to age, antibody waning, immune status) now have a larger effect on VE.

**Table 1:** A second initial analysis of the Legacy study (University College London Hospital and the Francis Crick Institute)

				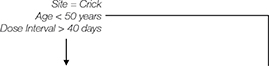				
	AZD1222			BNT162b2 (Cohort-Matched Subset)		BNT162b (Wall, Wu et al., Lancet, 2021)		
	Unique Participants n = 106		P-Value			Unique Participants n = 250		P-Value
	First Dose	Second Dose		Second Dose	P_(vs ChAdOx)_	First Dose	Second Dose	
	*Mean/Count (SD/%)*	*Mean/Count (SD/%)*		*Mean/Count (SD/%)*		*Mean/Count (SD/%)*	*Mean/Count (SD/%)*	
	n = 50	n = 63		n = 58		n = 149	n = 159	
*Site*			0.87		0.34			0.052
Crick	49 (98%)	62 (98.4%)		58 (100%)		95 (63.8%)	84 (52.8%)	
UCLH	1 (2%)	1 (1.6%)		0 (0%)		54 (36.2%)	75 (47.2%)	
*Age*			0.23		0.74			0.812
	37.3 (8.6)	35.3 (8.7)		35.8 (7.8)		42.7 (11.9)	43.1 (11.6)	
*Sex*			0.62		0.14			0.041
Female	34 (68%)	40 (63.5%)		44 (75.9%)		109 (73.2%)	99 (62.3%)	
Male	16 (32%)	23 (36.5%)		14 (24.1%)		40 (26.8%)	60 (37.7%)	
*BMI*			0.68		0.60			0.870
	23.8 (3.9)	23.5 (3.8)		23.7 (5.1)		24.9 (5.4)	24.9 (5.6)	
*Ethnicity (Grouped)*			0.04		0.13			0.362
All White Bkgs.	42 (84%)	42 (66.7%)		47 (81%)		123 (82.6%)	125 (78.6%)	
All S. Asian Bkgs.	0 (0%)	9 (14.3%)		2 (3.4%)		5 (3.4%)	11 (6.9%)	
All Other Bkgs.	7 (14%)	11 (17.5%)		9 (15.5%)		21 (14.1%)	23 (14.5%)	
(No response)	1 (2%)	1 (1.6%)		0 (0%)				

**Table 2:** Ordered logistic regression model and ANOVA of effect of strain and vaccine type on neutralising antibody response following 2-dose vaccination (Relates to Figure 1B).

Ordered Logistic Regression IC50_binned ~ Strain * VaccineType				
Factor	Coef.	S.E.	Wald Z	Pr(>|Z|)
**Strain (vs. Wildtype)**				
D614G	-2.5880	0.3894	-6.65	<0.0001
B.1.1.7	-1.6845	0.3816	-4.41	<0.0001
B.1.351	-3.5941	0.4058	-8.86	<0.0001
B.1.617.2	-3.7118	0.4000	-9.28	<0.0001
**Vaccine Type (vs. AZD1222)**				
BNT162b2	1.1504	0.3663	3.14	0.0017
Interactions (Strain * Vaccine Type)				
D614G * BNT162b2	1.3893	0.4902	2.83	0.0046
B.1.1.7 * BNT162b2	0.1391	0.4802	0.29	0.7721
B.1.351 * BNT162b2	1.1529	0.4951	2.33	0.0199
B.1.617.2 * BNT162b2	0.7467	0.4872	1.53	0.1254

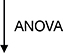			
Wald Statistics Response: IC50_binned			
Factor	Chi-Square	d.f.	P
**Strain (incl. Higher Order Factors)**	230.21	8	<.0001
**Vaccine Type (incl. Higher Order Factors)**	144.86	5	<.0001
**Interaction**	13.00	4	0.0113

**Table 3:** Ordered logistic regression model and ANOVA of effect of strain and self-reported prior COVID symptoms on neutralising antibody response following 1-dose AZD1222 vaccination (Relates to Figure 1D).

Ordered Logistic Regression IC50_binned ~ Strain * PriorCOVIDsymptoms				
Factor	Coef.	S.E.	Wald Z	Pr(>|Z|)
**Strain (vs. Wildtype)**				
D614G	-0.9181	0.4349	-2.11	0.0347
B.1.1.7	-1.7536	0.4695	-3.74	0.0002
B.1.351	-3.1014	0.6196	-5.01	<0.0001
B.1.617.2	-3.0659	0.6206	-4.94	<0.0001
**Prior COVID Symptoms (vs. those without)**				
With COVID Symptoms	2.0380	0.6474	3.15	0.0016
Interactions (Strain * Symptoms)				
D614G * Symptoms	0.7486	0.9235	0.81	0.4175
B.1.1.7 * Symptoms	1.4876	0.9444	1.58	0.1152
B.1.351 * Symptoms	2.4180	1.0096	2.39	0.0166
B.1.617.2 * Symptoms	1.9735	1.0019	1.97	0.0489

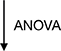			
Wald Statistics Response: IC50_binned			
Factor	Chi-Square	d.f.	P
**Strain (incl. Higher Order Factors)**	44.35	8	<.0001
**COVID Symptoms (incl. Higher Ord. Factors)**	89.98	5	<.0001
**Interaction**	7.55	4	0.1096

## Data Availability

All data (anonymised) and full R code to produce all figures and statistical analysis presented in this manuscript are freely-available online on Github: https://github.com/davidlvb/Crick-UCLH-Legacy-AZ-VOCs-2021-06
