# Supplementary material for: Ability of AZD1222 vaccination to elicit neutralising antibodies against SARS-CoV-2 VOC B.1.617.2 (Delta)
Source: Lancet. Author manuscript; Available in PMC 2021 Sep 17. (PMC8238446; doi:10.1016/S0140-6736(21)01462-8)
Supplement: Appendix [file EMS134648-supplement-Appendix.pdf]

Appendix for

Ability of AZD1222 vaccination to elicit neutralising antibodies against SARS-CoV-2 VOC B.1.617.2 (Delta)

Emma C Wall*[1,2], Mary Wu*[1], Ruth Harvey*[3], Gavin Kelly[1], Scott Warchal[1], Chelsea Sawyer[1], Rodney Daniels[3], Lorin Adams[3], Philip Hobson[1], Emine Hatipoglu[4], Yenting Ngai[4], Saira Hussain[1], Karen Ambrose[1], Steve Hindmarsh[1], Rupert Beale[1,2,6], Andrew Riddell[1], Steve Gamblin[1], Michael Howell[1], George Kassiotis[1,5], Vincenzo Libri[2,4] Bryan Williams [2,4], Charles Swanton[1,4], Sonia Gandhi*[1,4], David LV Bauer*[1,6]

Affiliations

1. The Francis Crick Institute, 1 Midland Road, London, UK
2. National Institute for Health Research (NIHR) University College London Hospitals (UCLH) Biomedical Research Centre and NIHR UCLH Clinical Research Facility, London, UK
3. Worldwide Influenza Centre, The Francis Crick Institute, 1 Midland Road, London, UK
4. University College London, Gower Street, London
5. Department of Infectious Disease, St Mary's Hospital, Imperial College London, London, UK
6. Genotype-to-Phenotype UK National Virology Consortium (G2P-UK)

*These authors contributed equally to this work.

Lead Authors: George Kassiotis George.Kassiotis@crick.ac.uk, Charles Swanton charles.swanton@crick.ac.uk, Sonia Gandhi sonia.gandhi@crick.ac.uk, and David LV Bauer david.bauer@crick.ac.uk

Correspondence to: David LV Bauer david.bauer@crick.ac.uk

Contents

Figures 1-4

Tables 1-3

Methods

Methods

Clinical cohort

Two prospective cohorts of Legacy participants were established in January 2021 (NCT04750356). Participants were included if they were an employee of either UCLH or the Francis Crick Institute and had submitted at least one sample for RT-qPCR occupational health testing for COVID-19 using the Crick testing pipeline. Participants consisted of patient-facing healthcare workers at UCLH, who had received at least one dose of a currently licensed COVID-19 vaccine and Crick staff. Participants were sampled at approximately 3 weeks post-vaccination and invited for follow-up visits at approximately 6 and 12 weeks. All participants were sampled at each visit with additional nasopharyngeal RT-qPCR for SARS CoV-2 (in addition to their occupational health testing) to exclude concurrent active infection, blood was collected for serological assays. Participants were analysed by vaccine type, vaccine dose number, date since vaccine dose, and self-reported prior COVID-19 symptoms.

Serological Analysis and Live-virus Neutralisation

All serological analysis, including live-virus neutralisation assay, were performed exactly as previously described (**Wall, Wu et al., Lancet, 2021**)

Data analysis, statistics

Study data were collected and managed using REDCap electronic data capture tools hosted at University College London[8,9]. Data were exported from REDCap into R for visualisation and analysis. IC50 values above the quantitative limit of detection of the assay (>2560) were recoded as 5120; IC_{50} values below the quantitative limit of the assay (< 40) but within the qualitative range were recoded as 10 and data below the qualitative range (i.e. no response observed) were recoded as 5. These changes do not affect any statistical parameters considered in the analysis and we do not perform analyses that consider that consider the absolute value of the points – i.e. rank-based analyses are used instead: statistical significance of the difference in median viral neutralisation IC_{50} values between different strains was performed using a paired Wilcoxon Ranked sum test. *p*-values reported have not been corrected for multiple testing. Fold-changes in median NAbTs (and 95% confidence intervals) between BNT162b2 and AZD1222 cohorts were determined using bootstrap statistics using the '*boot'* package in R, specifying vaccine type using the *strata* option. All graphs were generated using the '*ggplot2'* package. Analyses of stratified NAb responses by strain, vaccine type, and COVID symptoms of participants, were carried out using the *prop.test* function of the '*stats'* package in R, and ordered logistic regression using the *lrm* function of

the Regression Modeling Strategies (‘*rms*’) package in R, using the formula IC50 ~Strain*VaccineType or IC50 ~Strain*COVIDsymptoms , and *p*-values were calculated using the Wald Chi-Square test. Analysis of variance was carried out using the *anova* function in R.

Data Sharing

All data (anonymised) and full R code to produce all figures and statistical analysis presented in this manuscript are freely-available online on Github: https://github.com/davidlvb/Crick-UCLH-Legacy-AZ-VOCs-2021-06

Ethics

The Legacy study was approved by London Camden and Kings Cross Health Research Authority (HRA) Research and Ethics committee (REC) IRAS number 286469 and sponsored by University College London.

Role of the funding source

This work was undertaken at UCLH/UCL who received a proportion of funding from the National Institute for Health Research (NIHR) University College London Hospitals Department of Health’s NIHR Biomedical Research Centre (BRC). EW, VL and BW are supported by the Centre’s funding scheme. This work was supported jointly by the BRC and core funding from the Francis Crick Institute, which receives its funding from Cancer Research UK, the UK Medical Research Council, and the Wellcome Trust. DLVB is additionally supported by the Genotype-to-Phenotype National Virology Consortium (G2P-UK) via UK Research and Innovation and the UK Medical Research Council. The funders of the study had no role in study design, data collection, data analysis, data interpretation, or writing of the report. The corresponding authors had full access to all the data and the final responsibility to submit for publication.

Contributors Statement

Emma C Wall - Investigation, Data Curation, Writing - original draft. Has access to & has verified underlying data.
Mary Wu - Investigation, Methodology, Resources, Writing – review & editing, Conceptualization
Ruth Harvey - Investigation, Methodology, Resources, Writing – review & editing, Conceptualization
Gavin Kelly - Formal Analysis, Validation
Scott Warchal - Software, Methodology, Formal Analysis
Chelsea Sawyer - Software, Methodology, Formal Analysis
Rodney Daniels - Investigation
Lorin Adams - Investigation
Philip Hobson - Investigation
Emine Hatipoglu - Project administration, Conceptualization
Yenting Ngai - Project administration, Conceptualization
Saira Hussain - Investigation, Resources
Karen Ambrose - Supervision, Software, Methodology

Supplementary References

1. Ng KW, Faulkner N, Cornish GH, et al. Preexisting and de novo humoral immunity to SARS-CoV-2 in humans. *Science* 2020; **370**(6522): 1339-43.

2. Houlihan CF, Vora N, Byrne T, et al. Pandemic peak SARS-CoV-2 infection and seroconversion rates in London frontline health-care workers. *Lancet* 2020; **396**(10246): e6-e7.

3. Brown JC, Goldhill DH, Zhou J, et al. Increased transmission of SARS-CoV-2 lineage B.1.1.7 (VOC 2020212/01) is not accounted for by a replicative advantage in primary airway cells or antibody escape. *bioRxiv* 2021: 2021.02.24.432576.

4. Cele S, Gazy I, Jackson L, et al. Escape of SARS-CoV-2 501Y.V2 from neutralization by convalescent plasma. *Nature* 2021; **593**(7857): 142-6.

5. Faulkner N, Ng KW, Wu M, et al. Reduced antibody cross-reactivity following infection with B.1.1.7 than with parental SARS-CoV-2 strains. *bioRxiv* 2021: 2021.03.01.433314.

6. Rihn SJ, Merits A, Bakshi S, et al. A plasmid DNA-launched SARS-CoV-2 reverse genetics system and coronavirus toolkit for COVID-19 research. *PLoS Biol* 2021; **19**(2): e3001091.

7. van den Brink EN, Ter Meulen J, Cox F, et al. Molecular and biological characterization of human monoclonal antibodies binding to the spike and nucleocapsid proteins of severe acute respiratory syndrome coronavirus. *J Virol* 2005; **79**(3): 1635-44.

8. Harris PA, Taylor R, Minor BL, et al. The REDCap consortium: Building an international community of software platform partners. *J Biomed Inform* 2019; **95**: 103208.

9. Harris PA, Taylor R, Thielke R, Payne J, Gonzalez N, Conde JG. Research electronic data capture (REDCap)--a metadata-driven methodology and workflow process for providing translational research informatics support. *J Biomed Inform* 2009; **42**(2): 377-81.
